# Increase in Weight in Low Birth Weight and Very Low Birth Weight Infants Fed Fortified Breast Milk versus Formula Milk: A Retrospective Cohort Study

**DOI:** 10.3390/nu9050520

**Published:** 2017-05-20

**Authors:** Kris Yuet Wan Lok, Pui Hing Chau, Heidi Sze Lok Fan, Kam Ming Chan, Bill H. Chan, Genevieve P. C. Fung, Marie Tarrant

**Affiliations:** 1 School of Nursing, Li Ka Shing Faculty of Medicine, University of Hong Kong, 21 Sassoon Road, Pokfulam, Hong Kong SAR, China; phchau@graduate.hku.hk (P.H.C.); heidifsl@hku.hk (H.S.L.F.); marie.tarrant@ubc.ca (M.T.); 2 Department of Paediatrics & Adolescent Medicine, United Christian Hospital, 130 Hip Wo Street, Kwun Tong, Kowloon, Hong Kong SAR, China; chankm2@ha.org.hk (K.M.C.); chanhb@ha.org.hk (B.H.C.); fungpg@ha.org.hk (G.P.C.F.)

**Keywords:** preterm infants, human milk, nutrition, early neonatal growth, formula

## Abstract

There has been a dramatic rise in preterm births in developed countries owing to changes in clinical practices and greater use of assisted reproductive techniques. However, few studies have examined the growth and outcomes of preterm infants according to the type of feeding (with fortified breast milk or formula). The purpose of this study was to examine the effect of breast milk feedings and formula on the growth and short-term outcomes of preterm infants in Hong Kong. In a single-center retrospective cohort study, we included 642 preterm infants at gestational age <37 weeks with birth weights <2200 g. According to World Health Organization criteria, 466 were classified as low birth weight (LBW) infants (≥1500 g and <2200 g) and 176 were classified as very low birth weight (VLBW) infants (<1500 g). The mothers of approximately 80% of VLBW infants and 60% LBW infants initiated breast milk feeding. When compared with no breast milk intake, LBW infants that received breast milk were significantly more likely to have growth z-scores closer to the median of the reference population on admission and experienced slower weight gain from birth to discharge. When breast milk was categorized by percent of total enteral intake, significant differences were seen among LBW infants, with lower percentages of small-for-gestational-age (SGA) status at discharge with increased proportions of breast milk intake. Our results suggest that LBW infants fed breast milk had better growth z-scores and lower SGA status at discharge compared with those predominately fed preterm formula.

## 1. Introduction

Preterm birth is among the single conditions with the highest mortality and considerable risk of lifelong impairment [1]. Of the 135 million live births worldwide in 2010, around 15 million babies were born preterm, representing a global average preterm birth rate of 11.1% [2]. Today, preterm births occur in high-income countries and contribute substantially to neonatal mortality and morbidity [2]. Preterm birth rates represent 12.0% and 7.8% in the United States (US) and China, respectively [2]. Of the estimated 1.2 million preterm births in high-income countries, more than 0.5 million (42%) are in the United States [2]. China is ranked second of the 10 countries with the highest number of preterm births and the United States is ranked sixth [2].

Possible reasons for the dramatic rise in preterm births in high-income countries include older age during pregnancy, better access to infertility treatments, multiple pregnancies, and changes in obstetric practices [3]. Substantial preterm risk differences are evident between ethnic and racial groups. The role of ethnicity in preterm birth has been widely debated, but this variation has been linked to socioeconomic, lifestyle, and genetic factors [1]. Researchers have found that African-American infants tend to be born earlier than white infants [4,5] but are less likely to require special care [4]. A previous study in Hong Kong investigated the rate of preterm births (7.4%) and associated risk factors [6] and found that premature delivery was common among the Chinese population; they also found that hypertensive disorders of pregnancy, gestational diabetes, antepartum hemorrhage, and congenital malformations were significant risk factors for spontaneous preterm labor [6]. Substantial differences in preterm births among countries are evident; therefore, a better understanding of these high risk groups is required to improve care [1]. 

Infant feeding is a modifiable factor that can reduce mortality and disability owing to preterm birth. Researchers have shown that, among preterm infants, breastfeeding provides better health outcomes for both the infant and mother [7,8,9] when compared with infant formula feeding. It has been reported that premature infants who are breastfed have lower incidence of necrotizing enterocolitis (NEC) and late-onset sepsis and they have better feeding tolerance and neurodevelopmental outcomes [7,10,11,12]. The World Health Organization (WHO) recommends breast milk over formula for the initiation of feeds in premature infants. According to these guidelines, a mother should initiate pumping or breastfeeding within two hours after vaginal delivery and four hours after cesarean section [13].

It is difficult for preterm infants to be exclusively breastfed, however, owing to difficulty suckling at the breast and poor infant latch. Breast milk for preterm infants often requires nutrient fortification to meet their rapid growth and protein and mineral needs. Hence, enriched specialized preterm formula or donor milk is often used as an alternative if breastfeeding cannot be initiated. It has been well established that infants fed on donor milk had better outcomes than those fed on formula milk [14]. Meta-analysis has found that feeding with donor milk is associated with halving the risk of developing NEC compared with formula; however, donor milk may decrease the rate of short-term growth among low birth weight (LBW) infants [14]. Research from other countries has predominantly used data for fortified donor breast milk. Few studies have specifically examined the short-term outcomes of preterm infants fed fortified breast milk and formula milk during hospitalization. 

Further studies are warranted of breastfeeding initiation in preterm infants among women in Hong Kong. There is also limited data on breastmilk with fortification compared to formula and therefore additional data are needed in this area. Hong Kong can provide a unique focus for such studies because the city has no milk banks or donor milk as alternatives. This study aimed to extend the understanding of breastfeeding practices for preterm infants in Hong Kong. Specifically, this study aimed to evaluate short-term outcomes, such as growth among preterm infants, based on the type of feeding administered. 

## 2. Materials and Methods

### 2.1. Design, Setting, and Participants

We undertook a single center retrospective cohort study in a neonatal intensive care unit (NICU) at a major tertiary hospital in Hong Kong. From 2010–2014, we included preterm infants <37 weeks of gestational age with birth weights <2200 g. According to WHO definitions, we classified infants <1500 g as VLBW, and those ≥1500 g and <2200 g as LBW [15]. Clinical and demographic data such as maternal age, parity, delivery type, infant birth weight, birth length, birth head circumference, length of hospital stay, days of parenteral feeding, days of mechanical ventilation, and Apgar score were extracted from patient medical records during the first month of feeding. We concentrated on the first month because this period of life affects mostly short-term outcomes such as in-hospital growth (defined as change in weight z-score for gestational age), sepsis, and retinopathy of prematurity (ROP). For the purpose of the study, we designated neonatal feedings as “any breast milk” for feedings with breast milk and “no breast milk” as infant feedings with no breast milk (i.e., infant formula). Further analysis was conducted on growth outcomes by proportions of breast milk intake categorized by percent of total enteral intake during the first 30 days of hospitalization (<25%, 25%–50%, 50%–75%, and >75%). We excluded all infants who died, those with major congenital malformations or missing feeding data, and those who were transferred to other hospitals. Fenton growth z-scores were calculated for birth weight, discharge weight, and discharge head circumference [16]. Small-for-gestational-age (SGA) status at discharge was defined as <10th percentile for postmenstrual age according to the Fenton growth chart [16]. We chose to use z-scores rather than weight measurements because this allowed us to use hospital discharge as the outcome time point, which varies from subject to subject. It also provides standardization to a recognized scale, the widely used and validated Fenton growth chart [17]. Negative change in weight z-score from birth to discharge represents growth that is less than the predicted growth rate whereas positive change represents a growth rate greater than the predicted rate [17]. Data on linear growth were not reported owing to the fact that there were no length measurements taken during routine discharge from the NICU.

A pasteurized milk-based milk fortifier was added to meet the nutritional needs for human milk fortification. Trophic feedings were initiated 1–4 days after birth and were continued at 10–20 mL/kg/day as tolerated for up to 5 days. Subsequently, enteral nutrition volume was increased by 10–20 mL/kg/day. Decisions about increased in feeding volume and fortification were made by the clinical team based on the feeding guideline. The goals for enteral nutrition were 150 mL/kg/day and 120 kcal/kg/day. Maternal milk were fortified to 0.25–0.28g protein (3.5 kcal) (1 packet/100 mL of breast milk) when infants were tolerating at least 100 mL of milk intake per day.

Our primary aim was to examine the effect of feedings with breast milk and formula on the growth and short-term outcomes of preterm infants in Hong Kong. Secondary outcomes included hospital length of stay, sepsis, ROP, length of parenteral feeding, and days of mechanical ventilation.

### 2.2. Data Analysis

Fisher’s exact tests and *t*-tests were used to compare categorical variables and continuous variables, respectively, and to assess maternal and infant characteristics among milk intake groups. These analyses were repeated for proportions of milk intake according to four categories using chi-square or analysis of variance (ANOVA) tests, as appropriate. We used logistic regression models to explore the maternal and infant characteristics among milk intake groups. To explore the relationship between growth parameters (continuous variables) and milk intake group, linear regression was performed (Table S1). A 0.05 level of significance was used for the statistical analysis, and all data analyses were conducted using Stata version 14.1 statistical software (Stata Corp., College Station, TX, USA) [18]. 

### 2.3. Ethics

The study was approved by the University of Hong Kong-Hospital Authority Hong Kong West Cluster Joint Institutional Review Board (UW 15-037) and Hospital Authority Hong Kong Research Ethics Committee (Kowloon Central/East Cluster) (KC/KE-14-0230/ER-1).

## 3. Results

Between 1 January 2010, and 31 December 2014, a total 655 infants with birth weights <2200 g were admitted to a major tertiary NICU in Hong Kong. Of these, 6 infants died, 2 were transferred to other facilities, and 5 were excluded owing to missing data, leaving 642 participants (466 LBW, 176 VLBW) included in the analysis. From our study sample of preterm infants, 28% were SGA (<10th percentile for gestational age) at birth, and 51% were SGA at discharge. Approximately 60% and 80% of mothers initiated breastfeeding their LBW and VLBW infants, respectively.

Maternal and infant characteristics and short-term outcomes of participants are presented in Table 1. LBW infants that received breast milk feedings were more likely to be from mothers of older maternal age. When compared with those who had no breast milk intake, LBW infants that received breast milk were significantly more likely to have birth weights and length z-scores closer to the median of the reference population. However, when comparing short-term outcomes, LBW infants that received breast milk had significantly longer hospital lengths of stay and a higher number of days of parenteral feeding than those who received no breast milk. After adjusting for potential confounding variables (maternal age, delivery type, infant gender, sepsis, Apgar score <7 at 1 min and SGA at discharge), length of hospital stay and parenteral feeding days were insignificant to breast milk intake (Table S1).

The unadjusted odds ratios (ORs) and adjusted odds ratios (aORs) of receiving any breastmilk intake are presented in Table 2. Maternal age between 30–34 years (aOR 3.15; 95% confidence interval (CI), 1.33–7.45) or having a cesarean delivery (aOR 1.65; 95% CI, 1.02–2.68) was strongly associated with higher odds of receiving any breastmilk intake. On the contrary, small for gestational age at discharge was significantly associated with a lower odds of receiving any breastmilk intake (aOR, 0.43; 95% CI, 0.25–0.73).

Using the Fenton chart to calculate z-scores, no growth risks were identified in our study sample. Discharge weights among LBW infants were closer to the median of the reference population; i.e., growth was significantly better when fed breast milk during hospitalization compared with feeding preterm formula (Table 3). This pattern persisted when we classified the proportions of breast milk into four categories (Table 4). No significant differences in growth outcomes were observed among VLBW infants, except for a significantly slower gain in head circumference among VLBW infants fed any breast milk compared with those fed preterm formula. When we categorized growth outcomes by the proportion of breast milk intake, significantly slower weight and head circumference gains were found with increased proportions of breast milk intake. LBW infants fed any breast milk experienced a significantly larger decrease in weight z-score compared with LBW infants fed no breast milk (Table 4).

## 4. Discussion

In this retrospective study examining the effect of breast milk feeding compared with formula milk feeding on growth and short-term outcomes among preterm infants, no beneficial or adverse effects were found with breast milk on short-term outcomes such as sepsis, retinopathy, days of mechanical ventilation, and Apgar scores. Consistent with previous studies, we found an association between older maternal age, delivery type, and SGA at discharge influenced LBW infants receiving breast milk intake [19,20,21]. This reinforces the importance of providing breastfeeding support for those mothers who are either young or had vaginal delivery, or a small for gestational age infant at discharge may need special attention to establish successful breastfeeding among preterm births. Our preterm infant sample had good growth overall, according to Fenton z-score; LBW infants fed on breast milk had weight, length, and discharge weight z-scores that were significantly closer to the median of the population compared with those fed predominately preterm formula. We also found that our sample of LBW infants had lower percentages of SGA status at discharge with increased proportions of breast milk intake. However, LBW infants fed breast milk experienced slower weight gain between birth and discharge compared with formula fed infants. Consistent with other studies, preterm infants fed on breast milk or donor milk have significantly slower growth compared with those fed solely preterm formula [17,22]. A possible explanation from other studies is that slower growth rates found among infants fed fortified breast milk is owing to the inadequate protein content of standard fortified breast milk [17]. Currently, breastmilk composition is enhanced by adding a fixed dose fortification of breastmilk and this has been found to be insufficient in providing macronutrients at the recommended level in preterm infants [23,24]. Studies have shown that growth in preterm infants is linearly related to protein intake [25]. From our sample, although LBW infants had slower weight gains from birth to discharge, their overall discharge weight z-scores were significantly closer to the median weight of the population when compared with those predominately fed formula. This means that LBW infants fed fortified breast milk grew well, though at a slower rate compared with those fed formula milk. In comparison with the Hong Kong birth cohort, which is a large population representative of full-term Chinese infants, a majority of formula-fed infants had faster infant weight growth rates and higher body mass indexes at seven years of age [26]. Those authors concluded that faster growth may be indicative of good health status rather than being causally protective [26].

VLBW infants fed breast milk experienced growth trends similar to LBW infants, though these were not statistically significant. This could be owing to the small sample size of VLBW infants in our study. Future research with a larger sample size is necessary to more accurately assess this. From this study, observed differences seen could also be due to a reverse causation effect because mothers of more fragile infants are more likely to breastfeed owing to the potential benefit of better recovery; therefore, these infants could have longer lengths of stay and more parenteral feeding days. Thus, lengths of hospital stay and parenteral feeding days are not causally related to breast milk feedings. VLBW infants are more likely to receive breast milk, which suggests that mothers are more likely to breastfeed frailer infants.

Our study is the first to examine growth and short-term outcomes among Chinese preterm infants. However, there is a lack of standardization of methods used to calculate preterm infant growth velocity which makes comparisons between studies difficult [27,28]. The growth reference used also did not include Chinese infants in its development which make comparisons difficult. However, there is limited data on breastmilk with fortification compared to formula and this study further adds to the literature. The study benefited from a large, retrospectively collected data set of preterm infants that enabled comparisons of growth in terms of z-scores and short-term outcomes based on infant feeding intakes. Despite the large sample size, this was a single center study, and was limited in the small sample of VLBW infants. In addition, this was an observational study design, which may lead to residual confounding. We also extracted data for 30 days and further studies are warranted to address overall growth. From the extracted data, there is no information on how much time the infants were breastfed for. In addition, data on brain growth and neurological development is needed to assess somatic growth more accurately. Future study is warranted of different protein intakes or specific protein fortification of breast milk using a randomized study design, to further examine the association between different protein intakes and growth in preterm infants. We were also unable to assess the effects of fortified breast milk and formula milk intake on linear growth in our sample because infant length discharge data were not available. This is a major limitation of the study in not having length measurement to assess the infant growth. Future research using a larger sample size, multicenter neonatal units, and standardized length data can more accurately assess the impact of fortified breast milk on growth and short-term outcomes among preterm infants.

## 5. Conclusions

Overall, LBW infants fed breast milk had better growth z-scores and lower SGA status at discharge compared with those predominately fed preterm formula. The promotion and support of breastfeeding for LBW infants is important. More in-depth research is needed to identify the short-term outcomes associated with preterm infant feeding at multicenter neonatal units.

## Figures and Tables

**Table 1:** Characteristics of LBW and VLBW preterm infants breastfed or not breastfed.

	LBW			VLBW		
	No Breastmilk (*n* = 190) *n* (%)	Any Breastmilk (*n* = 276) *n* (%)	*p*-Value	No Breastmilk (*n* = 31) *n* (%)	Any Breastmilk (*n* = 145) *n* (%)	*p*-Value
Characteristics of mothers						
Maternal age (years)			0.022			0.702
18–24	30 (15.8)	22 (8.0)		3 (9.7)	15 (10.3)	
25–29	36 (19.0)	49 (17.8)		7 (22.6)	26 (17.9)	
30–34	56 (29.5)	110 (39.9)		12 (38.7)	47 (32.4)	
>35	68 (35.8)	95 (34.4)		9 (29.0)	57 (39.3)	
Parity			0.183			0.322
Primiparous	101 (47.6)	165 (59.8)		16 (51.6)	89 (61.4)	
Multiparous	88 (46.6)	111 (40.2)		15 (48.4)	56 (38.6)	
Delivery type			0.089			0.421
Vaginal delivery	99 (52.1)	121 (43.8)		10 (32.3)	60 (41.4)	
Cesarean delivery	91 (47.9)	155 (56.2)		21 (67.7)	85 (58.6)	
Induced labour						1.000
No	174 (91.6)	260 (94.2)	0.271	31 (100.0)	143 (98.6)	
Yes	16 (8.4)	16 (5.8)		0 (0.0)	2 (1.4)	
Characteristics of newborn						
Gender			0.257			0.101
Male	100 (52.6)	160 (58.0)		15 (48.4)	95 (65.5)	
Female	90 (47.4)	116 (42.0)		16 (51.6)	50 (34.5)	
Birthweight (g), mean (SD)	1940.0 (186.5)	1875.4 (199.9)	<0.001 ^a^	1269.3 (180.6)	1139.2 (205.9)	<0.001 ^a^
Birthweight z score, mean (SD)	−0.89 (0.9)	−0.61 (0.9)	<0.001 ^a^	−0.69 (0.9)	−0.71 (1.0)	0.930 ^a^
SGA at birth	68 (35.8)	63 (22.8)	0.002	7 (23.3)	43 (29.9)	0.516
Birth length (cm), mean (SD)	43.4 (2.3)	43.2 (2.6)	0.453 ^a^	38.3 (2.5)	37.1 (2.9)	0.038 ^a^
Birth length z score, mean (SD)	−0.67 (1.1)	−0.33 (1.1)	0.002 ^a^	−0.73 (1.1)	−0.76 (1.4)	0.901 ^a^
Birth head circumference (cm), mean (SD)	30.4 (1.4)	30.0 (1.5)	0.002 ^a^	26.7 (1.4)	26.4 (1.7)	0.389 ^a^
Sepsis			0.249			1.000
No	189 (99.5)	268 (97.8)		30 (96.8)	137 (95.8)	
Yes	1 (0.5)	6 (2.2)		1 (3.2)	6 (4.2)	
Retinopathy			0.272			0.055
No	190 (100.0)	269 (98.9)		26 (83.9)	94 (65.7)	
Yes	0 (0.0)	3 (1.1)		5 (16.1)	49 (34.3)	
Length of stay (days)			0.005 ^a^			0.617 ^a^
Mean (SD)	20.0 (13.8)	24.8 (20.5)		87.9 (148.8)	78.6 (78.0)	
Parenteral feeding days			0.026 ^a^			0.671 ^a^
Mean (SD)	0.8 (3.9)	2.1 (7.3)		19.0 (17.0)	17.9 (11.7)	
Mechanical ventilation days			0.274 ^a^			0.071 ^a^
Mean (SD)	0.1 (0.7)	0.2 (2.6)		1.7 (3.3)	5.3 (10.9)	
Apgar score <7 at 1 min, mean (SD)	11 (8.6)	13 (6.7)	0.523	2 (6.9)	20 (14.7)	0.372
Apgar score <7 at 5 min, mean (SD)	0 (0.0)	1 (1.2)	1.000	0 (0.0)	4 (4.0)	1.000

*p* value refers to that from Fisher’s exact test unless otherwise stated. ^a^ Independent *t*-test.

**Table 2:** Unadjusted and adjusted odds ratios for breastfed infants by participants’ characteristics.

	LBW				VLBW			
	OR	95% CI	aOR	95% CI	OR	95% CI	aOR	95% CI
Maternal age								
18–24 years	1.00	-	1.00	-	1.00	-	1.00	-
25–29 years	1.86	0.92–3.73	2.80	1.10–7.18	0.74	0.17–3.31	0.55	0.11–2.82
30–34 years	2.68	1.42–5.07	3.15	1.33–7.45	0.78	0.19–3.15	0.79	0.18–3.56
≥35 years	1.91	1.01–3.58	2.46	1.04–5.84	1.27	0.30–5.27	1.23	0.28–5.50
Parity								
Primiparous	1.00	-	1.00	-	1.00	-	1.00	-
Multiparous	0.77	0.53–1.12	0.63	0.39–1.04	0.67	0.31–1.46	0.57	0.23–1.37
Delivery type								
Vaginal delivery	1.00	-	1.00	-	1.00	-	1.00	-
Cesarean delivery	1.39	0.96–2.01	1.65	1.02–2.68	0.67	0.30–1.54	0.55	0.21–1.44
Infant gender								
Male	1.00	-	1.00	-	1.00	-	1.00	-
Female	0.81	0.56–1.17	0.68	0.42–1.10	0.49	0.23–1.08	0.48	0.20–1.18
Sepsis								
No	1.00	-	1.00	-	1.00	-	1.00	-
Yes	4.23	0.51–35.43	4.01	0.44–36.81	1.31	0.15–11.31	1.29	0.13–12.55
Apgar score <7 at 1 min								
No	1.00	-	1.00	-	1.00	-	1.00	-
Yes	0.76	0.33–1.75	0.63	0.26–1.54	2.33	0.51–10.56	2.17	0.46–10.29
SGA at discharge								
No	1.00	-	1.00	-	1.00	-	1.00	-
Yes	0.53	0.35–0.80	0.43	0.25–0.73	1.40	0.56–3.50	2.77	0.88–8.71

Adjusted for maternal age, parity, delivery type, infant gender, sepsis, Apgar score <7 at 1 min and SGA at discharge.

**Table 3:** Growth outcomes by breastmilk intake.

**LBW**	**All Subjects (*n* = 466)**	**No Breastmilk (*n* = 190)**	**Any Breastmilk (*n* = 276)**	***p*-Value**
Discharge weight (g), mean (SD)	2364.3 (461.2)	2332.3 (386.6)	2386.4 (505.8)	0.214
Discharge weight z-score, mean (SD)	−1.26 (0.9)	−1.38 (0.8)	−1.19 (0.9)	0.017
Change in weight z score, birth to discharge, mean (SD)	−0.54 (0.55)	−0.48 (0.58)	−0.58 (0.52)	0.070
Discharge head circumference (cm), mean (SD)	32.0 (1.6)	31.9 (1.3)	32.0 (1.8)	0.279
Change in head circumference z score, mean (SD)	−0.77 (0.9)	−0.90 (0.9)	−0.69 (0.9)	0.022
SGA at discharge *n* (%)	239 (51.4)	103 (54.2)	136 (49.5)	0.313 ^a^
**VLBW**	**All Subjects (*n* = 175)**	**No Breastmilk (*n* = 31)**	**Any Breastmilk (*n* = 144)**	***p*-Value**
Discharge weight (g), mean (SD)	2941.3 (1135.8)	3043.4 (1522.2)	2919.3 (1135.8)	0.583
Discharge weight z-score, mean (SD)	−1.37 (1.3)	−1.26 (1.3)	−1.39 (1.3)	0.623
Change in weight z score, birth to discharge, mean (SD)	−0.66 (1.1)	−0.55 (1.0)	−0.68 (1.1)	0.541
Discharge head circumference (cm), mean (SD)	33.5 (2.5)	32.9 (1.7)	33.6 (2.6)	0.164
Change in head circumference z score, mean (SD)	−0.90 (1.1)	−0.64 (1.2)	−0.95 (1.1)	0.201
SGA at discharge *n* (%)	89 (51.2)	11 (36.7)	78 (54.2)	0.081 ^a^

^a^ Independent *t*-test.

**Table 4:** Growth outcomes by proportion of breastmilk intake.

**LBW**	**All Subjects (*n* = 466)**	**Group by Proportions of Breastmilk Intake**				***p*-Value for All Groups**
		**<25%**	**25%–50%**	**50%–75%**	**>75%**	
Birthweight (g), mean (SD)	1902 (196.9)	1930 (187.9)	1819 (223.3)	1808 (188.0)	1742 (151.0)	<0.001
Birthweight z-score, mean (SD)	−0.72 (0.89)	−0.81 (0.89)	−0.53 (0.90)	−0.29 (0.82)	−0.39 (0.80)	<0.001
Discharge weight (g), mean (SD)	2364 (461.2)	2345 (469.5)	2530 (546.3)	2361 (289.0)	2419 (371.5)	0.133
Discharge weight z-score, mean (SD)	−1.26 (0.86)	−1.32 (0.82)	−1.07 (1.17)	−1.04 (0.84)	−0.98 (0.88)	0.029
Change in weight z score, birth to discharge, mean (SD)	−0.54 (0.55)	−0.52 (0.52)	−0.54 (0.85)	−0.75 (0.36)	−0.60 (0.62)	0.109
Discharge head	32	32	32	32	33	0.036
Circumference (cm)	(1.62)	(1.62)	(1.60)	(1.06)	(2.02)	
Change in head	−0.30	−0.31	−0.18	−0.54	0.08	0.080
circumference, mean (SD)	(0.90)	(0.81)	(1.07)	(0.80)	(1.68)	
SGA at discharge n (%)	239 (51.4)	201 (54.5)	15 (44.1)	16 (47.1)	7 (25.0)	0.020
**VLBW**	**All Subjects (*n* = 175)**	**Group by Proportions of Breastmilk Intake**				***p*-Value for All Groups**
		**<25%**	**25%–50%**	**50%–75%**	**>75%**	
Birthweight (g), mean (SD)	1162 (207.2)	1213 (204.3)	1202 (189.5)	1135 (221.5)	1106 (191.4)	0.033
Birthweight z-score, mean (SD)	−0.71 (0.99)	−0.85 (0.99)	−0.99 (0.98)	−0.56 (0.83)	−0.53 (1.12)	0.125
Discharge weight (g), mean (SD)	2941 (1135.8)	2975 (1276.3)	2958 (1228.7)	2914 (868.8)	2920 (1177.6)	0.992
Discharge weight z-score, mean (SD)	−1.37 (1.28)	−1.17 (1.43)	−1.31 (1.32)	−1.36 (1.08)	−1.63 (1.25)	0.342
Change in weight z score, birth to discharge, mean (SD)	−0.66 (1.07)	−0.32 (1.22)	−0.32 (0.64)	−0.80 (0.87)	−1.10 (1.09)	<0.001
Discharge head	34	33	33	34	33	0.985
Circumference (cm)	(2.53)	(2.83)	(2.84)	(2.16)	(2.40)	
Change in head	−0.21	0.35	0.02	−0.44	−0.77	0.011
circumference, mean (SD)	(1.70)	(1.34)	(1.01)	(1.36)	(2.39)	
SGA at discharge n (%)	89 (51.2)	23 (41.8)	13 (52.0)	23 (48.9)	30 (63.8)	0.169

## Supplementary Materials

The following are available online at www.mdpi.com/2072-6643/9/5/520/S1, Table S1: Adjusted linear regression between breastfed infants and growth parameters.
