# Supplementary material for: Increase in Weight in Low Birth Weight and Very Low Birth Weight Infants Fed Fortified Breast Milk versus Formula Milk: A Retrospective Cohort Study
Source: Nutrients. 2017 May 20;9(5):520. doi: 10.3390/nu9050520 (PMC5452250; doi:10.3390/nu9050520)
Supplement: Supplementary file 1 [file nutrients-09-00520-s001.docx]

**Table S1**. Adjusted linear regression between breastfed infants and growth parameters.

| **Outcomes** | **LBW (*n* = 466)** | | | **VLBW (*n* = 176)** | | |
| --- | --- | --- | --- | --- | --- | --- |
|  | **Mean Difference** | **95% CI** | ***p*-Value** | **Mean Difference** | **95% CI** | ***p*-Value** |
| Birthweight (g) | –38.88 | –72.25, –5.50 | 0.023 | –118.87 | –191.78, –45.96 | 0.002 |
| Birth weight z score | 0.28 | 0.12, 0.45 | 0.001 | –0.13 | –0.47, 0.21 | 0.443 |
| Birth length (cm) | –0.04 | –0.52, 0.44 | 0.871 | –0.87 | –2.01, 0.26 | 0.130 |
| Birth length z score | 0.34 | 0.12, 0.56 | 0.003 | –0.03 | –0.55, 0.49 | 0.909 |
| Birth head circumference (cm) | –0.34 | –0.59, –0.08 | 0.009 | –0.35 | –1.01, 0.30 | 0.289 |
| Discharge weight (g) | –49.38 | –88.66, –10.09 | 0.014 | –68.96 | –292.22, 154.30 | 0.543 |
| Discharge weight z score | 0.18 | 0.02, 0.34 | 0.026 | –0.15 | –0.64, 0.34 | 0.553 |
| Discharge head circumference (cm) | –0.11 | –0.36, 0.15 | 0.411 | –0.05 | –0.78, 0.68 | 0.896 |
| Discharge head circumference z score | 0.20 | 0.02, 0.38 | 0.031 | –0.12 | –0.60, 0.37 | 0.634 |
| Adjusted for infant gender, maternal age, parity, delivery type, length of hospital stay, parenteral feeding days and mechanical ventilation days | | | | | | |
| Length of stay (days) ^a^ | 2.28 | –0.06, 4.62 | 0.056 | 3.23 | –24.42, 30.88 | 0.818 |
| Parenteral feeding days ^b^ | 0.60 | –0.27, 1.48 | 0.177 | –3.72 | –8.15, 0.71 | 0.099 |
| Mechanical ventilation days ^c^ | –0.05 | –0.17, 0.06 | 0.356 | 3.64 | 0.33, 6.95 | 0.031 |

^a^ Adjusted for infant gender, maternal age, parity, delivery type, parenteral feeding days and mechanical ventilation days; ^b^ Adjusted for infant gender, maternal age, parity, delivery type, length of stay and mechanical ventilation days; ^c^ Adjusted for infant gender, maternal age, parity, delivery type, length of stay and parenteral feeding days.
